# How I prescribe prolonged intermittent renal replacement therapy

**DOI:** 10.1186/s13054-023-04389-7

**Published:** 2023-03-08

**Authors:** Edward G. Clark, Anitha Vijayan

**Affiliations:** 1 Division of Nephrology, Department of Medicine, University of Ottawa, Ottawa, Canada; 2 Division of Nephrology, Washington University in St. Louis, St. Louis, MO USA

## Abstract

Prolonged Intermittent Renal Replacement Therapy (PIRRT) is the term used to define ‘hybrid’ forms of renal replacement therapy. PIRRT can be provided using an intermittent hemodialysis machine or a continuous renal replacement therapy (CRRT) machine. Treatments are provided for a longer duration than typical intermittent hemodialysis treatments (6–12 h vs. 3–4 h, respectively) but not 24 h per day as is done for continuous renal replacement therapy (CRRT). Usually, PIRRT treatments are provided 4 to 7 times per week. PIRRT is a cost-effective and flexible modality with which to safely provide RRT for critically ill patients. We present a brief review on the use of PIRRT in the ICU with a focus on how we prescribe it in that setting.

## Introduction

Prolonged Intermittent Renal Replacement Therapy (PIRRT) is the term that broadly encompasses ‘hybrid’ forms of renal replacement therapy (RRT). PIRRT treatments are provided for a longer duration than are intermittent hemodialysis (IHD) treatments (6–12 h vs. 3–4 h, respectively) but not 24 h per day as is done for continuous renal replacement therapy (CRRT). PIRRT is typically provided 4 to 7 times per week [1].

While PIRRT is less commonly used in ICUs than IHD or CRRT, its use has been progressively increasing in low- and middle-income countries [2, 3] since its initial descriptions in the literature in the late 1990s [4, 5]. Its routine use in some high-income countries (e.g., institutions in New Zealand [6] and Canada [7]) is also long-established. It is a cost-effective (as compared to CRRT [7, 8]) and flexible modality with which to safely provide RRT for hemodynamically unstable patients. During the COVID-19 pandemic, PIRRT was rapidly adopted at some institutions to maximize their acute RRT capacity during surge. [9–11].

### Indications for PIRRT

KDIGO 2012 guidelines state that CRRT is the treatment of choice for hemodynamically unstable patients, including those on extracorporeal support such as ECMO. However, at that time data on PIRRT were scarce. At present, PIRRT is used as a substitute for CRRT to treat hemodynamically unstable patients with acute kidney injury (AKI) or ESRD [12]; it can also be used in patients during de-escalation of treatment in the ICU [13], or as a substitute for IHD. Less well-studied than IHD or CRRT, there is no evidence suggesting significant differences in mortality or kidney recovery with the use of PIRRT to manage severe AKI in critically ill patients as compared to CRRT [14]. Reducing the efficiency of solute clearance (thereby reducing osmotic shifts) and extending the duration of treatment (thereby lowering the ultrafiltration rate) make PIRRT less likely to provoke hemodynamic instability during RRT (HIRRT) relative to IHD [15]. As an intermittent therapy, PIRRT facilitates the performance of diagnostic imaging, rehabilitation, and other procedures, and can often be provided overnight.

In certain situations, PIRRT is relatively contraindicated. For patients with intoxications or extreme electrolyte disturbances where highly efficient small molecule clearance is desired, IHD should be favored over PIRRT (or CRRT). Conversely, in patients with traumatic brain injury, increased intracranial pressure or severe hyponatremia, CRRT should be favored over PIRRT (or IHD).

### PIRRT modalities

PIRRT can be delivered using a standard IHD machine (with a connection to a central purified water-supply or the use of a portable/built-in reverse-osmosis machine) or a CRRT machine using standard commercially available CRRT solutions. In either case, adjustments are made to the blood flow rate (Qb), and dialyzate rate (Qd) and/or replacement fluid rates. These modifications are made to reduce the efficiency of solute clearance relative to standard IHD (and provide it for a longer duration) or increase clearance relative to CRRT (and provide it for a shorter duration). When using a conventional IHD machine to provide PIRRT, the machine software may not allow the Qd to be reduced enough to markedly decrease the efficiency of solute clearance. In such cases, a CRRT or pediatric IHD dialyzer (filter) with a relatively small surface area may be utilized to further reduce efficiency. Depending on the machines used and local experience, specific PIRRT modalities utilize diffusive clearance (i.e., hemodialysis; e.g., sustained low-efficiency (daily) dialysis [SLED/SLEDD]), convective clearance (i.e., hemofiltration; e.g., accelerated veno-venous hemofiltration [AVVH]) or both (i.e., hemodiafiltration; e.g., sustained low-efficiency (daily) diafiltration [SLED-f /SLEDD-f]).

### Vascular access

Vascular access considerations for patients with AKI are similar to when prescribing CRRT [16]. For patients with pre-existing kidney failure and an arteriovenous fistula (AVF) or arteriovenous graft (AVG), unless IHD-trained nurses are routinely involved in the provision of PIRRT and measures are in-place to prevent dislodgement of access needles, a hemodialysis catheter is required for PIRRT.

### Anticoagulation

There is less need for anticoagulation with the use of PIRRT compared with CRRT, largely due to the higher Qb. In the absence of another indication for anticoagulation, we prescribe PIRRT without any anticoagulation (i.e., saline flushes only). When anticoagulation is indicated due to issues with filter clotting or otherwise, unfractionated heparin is most commonly used. If CRRT machines are used to provide PIRRT and regional citrate anticoagulation is possible, it is the option of choice.

### Typical treatment parameters for PIRRT

Table 1 details sample PIRRT prescriptions according to whether a conventional IHD machine or a CRRT machine is being used and relative to standard IHD and CRRT treatments. Successful development and implementation of routine PIRRT protocols necessitate a collaborative approach. The input of nephrologists, critical care physicians, nurses, pharmacists and administrators is required.

**Table 1:** PIRRT Using IHD and CRRT Machines in Comparison with Standard IHD and CRRT Prescriptions

Parameter	Modality			
	Standard Intermittent IHD	PIRRT		Standard CRRT
		Using IHD Machine	Using CRRT Machine	
Clearance mode	Diffusion	Diffusion or Diffusion + Convection	Diffusion or Convection or Diffusion + Convection	
Blood flow rate	≥ 300 mL/min	100–300 mL/min		100–200 mL/min
Duration	3–4 h	6–12 h	8–12 h	Continuous
Frequency	3–4 days/week	4–7 days/week		
Dialyzate rate	500–800 mL/min	100–400 mL/min		10–30 mL/min
Replacement Rate*	N/A	1–2 L/hour	20–40 mL/min	10–30 mL/min
Dialyzer Surface area	1.0–2.5 m^2^	0.6–2.5 m^2^	0.6–1.5 m^2^	
Need for Anticoagulation	+	+		+ + +
Dialyzate [Na^+^]	145 mmol/L^δ^		140 mmol/L^Ψ^	
Dialyzate [K^+^]	4 mmol/L^δ^			
Dialyzate [Ca + +]	1.5 mmol/L^δ^		1.75 mmol/L (0 mmol/L if using RCA) ^Ψ^	
Dialyszte [HCO_3_]	24 – 36 mmol/L^δ^		32 mmol/L^Ψ^	
Options for pre-emptive PO_4_ supplementation once serum [PO_4_] ≤ 1–1.6 mmol/L	Add PO_4_ to dialyzate		Add PO_4_ to standard CRRT fluids or switch to commercially available PO_4_-containing fluids	
	Oral/enteral PO_4_ administration			

^*^Only applicable if convective clearance (hemofiltration) is being employed

^δ^Standard concentrations; may be adjusted as indicated clinically

^Ψ^Using commercially available solutions

[Na^+^], sodium concentration; [K^+^], potassium concentration; [Ca + +], calcium concentration; [HCO_3_], bicarbonate solution; RCA, regional citrate anticoagulation; [PO_4_], phosphate concentration

### Complications/safety

When ordering PIRRT that is delivered using a conventional IHD machine, use of a low dialyzate temperature (i.e., 35–35.5 °C) [17], relatively high dialyzate sodium and calcium concentrations (e.g., 145 mmol/L and 1.5 mmol/L, respectively) may help mitigate HIRRT [18]. In patients with significant hyponatremia (e.g., serum sodium ≤ 130 mmol/L), the dialyzate sodium should be reduced to a level that will prevent overly rapid correction assuming that equilibration between the serum and dialyzate sodium will occur before the end of treatment. When using a conventional IHD machine with on-line generation of dialyzate, dialyzate bicarbonate levels must also be reduced to allow for generation of dialyzate sodium concentrations at the lower end of what the machine allows (typically ~ 130 mmol/L). Similarly, when ordering dialyzate potassium concentration, it is safest to assume that complete equilibration will occur prior to the end of the treatment. Thus, unless the patient is profoundly hyperkalemic and/or more-rapid correction is mandated (i.e., serum potassium ≥ 6.5 mmol/L or acutely rising) then a dialyzate potassium of 4 mmol/L can be used routinely to avoid precipitating hypokalemia.

Hypophosphatemia is a frequent complication of any continuous or prolonged RRT and is often under recognized [19]. Hypophosphatemia during RRT can lead to tissue hypoxia [20] and is associated with prolonged ventilator dependence [21]. Pre-emptive management is key since effects of phosphate depletion can occur even without overt hypophosphatemia. At one author’s (AV) institution, the PIRRT protocol calls for starting oral supplementation when serum phosphate is less than 1.1 mmol/L. At the other author’s (EC) institution, where IHD equipment is used to provide PIRRT, a phosphate additive is routinely added to dialyzate when serum phosphate is less than 1.6 mmol/L. Other pre-emptive strategies include using phosphate-containing solutions (if CRRT equipment is used to provide PIRRT). Intravenous phosphate supplementation may be required for moderate to severe hypophosphatemia (< 0.6 mmol/L).

Antibiotic and other medication dosing data in PIRRT are limited and, ideally, should be considered in conjunction with the input of a critical care or nephrology pharmacist. For medications cleared during RRT, augmented or additional dosing may be required. For example, intravenous vancomycin may need to be given immediately before and after a 10–12 h PIRRT session to ensure an adequate therapeutic level during and post-treatment. Table 2 provides additional details regarding dosing of selected antibiotics in patients receiving PIRRT [22–28], a topic that has been explored in greater detail by other reviews [29, 30].

**Table 2:** Prescription of Selected Anti-infective Agents for Critically Ill Patients Receiving PIRRT

Anti-infective Agent [Relevant REFs]	Suggested Dosing Regimen*	Comments	
Vancomycin [22, 23]	Loading dose of 2400 mg then 1600 mg post-treatment	Clearance with PIRRT is ~ 3X higher than is described for CRRT	Ongoing dosing guided by post-PIRRT trough levels
Piperacillin [24, 25]	3 g every 8 h for susceptible organisms with MIC ≤ 16 mg/L OR 9 g dose as a continuous infusion every 24 h for susceptible organisms with MIC ≤ 32 mg/L		PIRRT reduces penicillin and carbapenem concentrations by approximately 50%. If pre-treatment concentration is ≥ 2X breakpoint of target attainment before treatment, subtherapeutic levels will generally be prevented
Meropenem [23, 25–27]	Maintenance dose of 1 g every 8 h or every 12 h	Wide variation across institutions; most frequently recommended regimen: 1 g every 12 h [26]	
Fluconazole [28]	Loading dose of 800 mg followed by 400 mg twice daily (q12h or pre- and post- PIRRT)	Recommendation based on Monte Carlo simulations using a pharmacokinetic model of PIRRT. Directly measured pharmacokinetic data for fluconazole (and most anti-infective agents) are limited in this setting	

REFs, references; MIC, minimum inhibitory concentration; PIRRT, prolonged intermittent renal replacement therapy; CRRT, continuous renal replacement therapy

*Suggested dosing is based on an assumption that PIRRT is provided as sustained low-efficiency dialysis using a dialyzer with a surface area of 0.7 m^2^, Blood Flow Rate (Qb) of 200 ml/min, Dialyzate Flow Rate (Qd) of 300 ml/min and prescribed as 8-h sessions once daily. Dosing regimens should be adjusted according to the relative efficiency/clearance mode(s) of PIRRT being provided, residual kidney function and other standard dosing considerations (e.g., patient weight, volume of distribution, etc.). A more detailed summary of anti-infective dosing studies across various forms of PIRRT can be found in other reviews [29, 30]. We suggest that all anti-infective agents for critically ill patients receiving PIRRT are prescribed in conjunction with a critical care pharmacist and guided by directly measured levels, whenever possible

### Dose/adequacy

Unlike dosing recommendations for CRRT and IHD (based on RENAL [31] and ATN [32] trials), there is no standard recommendation for dosing of PIRRT. Despite significant pitfalls in its use, urea kinetics remain the mainstay of determining adequacy of clearance during RRT, even in AKI. When prescribing PIRRT as a substitute for CRRT, a minimum weekly standard Kt/Vurea of 6 may be required. If using as a substitute for IHD or as a transition therapy, then lower flow rates or decreased frequency of treatments may suffice, as weekly standard Kt/Vurea recommendations for IHD is 2 [1]. It should be noted that volume overload is also an indication for RRT and frequency of PIRRT treatments ultimately will also depend on volume status and metabolic derangements such as hyperkalemia.

## Conclusions

The various forms of PIRRT used in ICU allow for cost-effective and flexible treatments for critically ill patients with kidney failure. As detailed in Table 1, practical considerations related to its application depend on whether IHD or CRRT machines are used to provide PIRRT. As is the case for our colleagues who prescribe CRRT [16], at institutions that provide PIRRT, we similarly advocate for its protocolized application accompanied by routine monitoring of quality and safety.

## Data Availability

Not applicable.
